# Alterations in cognitive function among reproductive‐age women with polyendocrine metabolic ovarian syndrome: A systematic review and meta‐analysis

**DOI:** 10.1111/jne.70225

**Published:** 2026-07-03

**Authors:** Samantha Rogers, Bailey Milne, Jennifer Carpenter

**Affiliations:** ^1^ Department of Health Sciences Queen's University Kingston Ontario Canada; ^2^ Department of Public Health Sciences Queen's University Kingston Ontario Canada; ^3^ Department of Emergency Medicine Queen's University Kingston Ontario Canada

**Keywords:** cognitive function, endocrine disorder, executive function, polyendocrine metabolic syndrome (PMOS)

## Abstract

Polyendocrine metabolic ovarian syndrome (PMOS) is a common endocrine disorder affecting 1.55 million women of reproductive age worldwide. While its physical and reproductive impacts are well‐documented, the cognitive effects of PMOS remain under‐researched and poorly understood. This systematic review aims to investigate existing literature on cognitive function in women with PMOS. The objective of this review is to identify specific cognitive differences between women with PMOS and those without, highlight gaps in current research and clinical care, and inform the development of more comprehensive care frameworks for individuals with PMOS. A systematic search was conducted using Ovid MEDLINE, Ovid EMBASE, and CINAHL (Cumulative Index to Nursing and Allied Health Literature) for studies published up to June 23, 2025. Eligible studies underwent data extraction and risk of bias assessment in accordance with standard systematic review protocols. Out of 2887 articles screened, 22 studies met the inclusion criteria. Twenty‐two studies involving 136,008 women with PMOS and 333,895 without PMOS were included. Across cognitive domains, pooled correlations generally indicated small, non‐significant negative associations, including executive function (*r* = −0.12, 95% confidence interval [CI] –0.26 to 0.02), attention (*r* = −0.13, 95% CI –0.38 to 0.14), and working memory (*r* = −0.14, 95% CI –0.31 to 0.05). Substantial heterogeneity was observed in several domains, often driven by individual outlying studies. Risk of bias was generally low, and funnel plots with Egger's regression provided no evidence of publication bias. This review underscores the need for further research into the cognitive aspects of PMOS. Addressing this gap is essential for developing a more comprehensive approach to PMOS treatment and support beyond its traditional focus on physical and reproductive health.

## 1 INTRODUCTION

Polyendocrine metabolic ovarian syndrome (PMOS) is the most common heterogeneous endocrine disorder among women.^1^ This syndrome causes dysregulation of reproductive hormones, including luteinizing hormone (LH), follicle‐stimulating hormone (FSH), estrogen, and testosterone, leading to multifaceted clinical symptoms.^2^ These symptoms typically emerge between the ages of 18 and 39 years and commonly include irregular menstrual cycles, often oligomenorrhea or secondary amenorrhea, hirsutism, acne, and high insulin levels.^3^, ^4^, ^5^ PMOS is more likely to develop in those with comorbidities such as obesity, type 2 diabetes, infertility, endometrial dysplasia, psychiatric disorders, and cardiovascular disease.^2^ PMOS affects an estimated 5%–26% of reproductive‐aged women worldwide, depending on the diagnostic criteria used.^2^, ^6^, ^7^ The Rotterdam criteria are the more widely used diagnostic method and require the presence of at least two of the following three features: ovulatory dysfunction, hyperandrogenism (clinical or biochemical), and polycystic ovarian morphology.^2^

Beyond its reproductive and metabolic manifestations, PMOS is also associated with adverse physical and psychological outcomes. Women with PMOS experience higher rates of adverse reproductive, cardiovascular, psychological, and metabolic outcomes than the general female population.^8^ They also have an increased likelihood of depression and anxiety, which significantly decreases quality of life.^3^ While the physical and psychological effects of PMOS have been widely studied, its impact on cognitive function is less certain. Existing studies suggest a consistent trend of reduced performance in women with PMOS on cognitive tasks, including executive functioning, attention, and working memory, compared to healthy controls; however, a consensus has not yet been reached.^9^, ^10^, ^11^, ^12^, ^13^ Thus, this systematic review aims to synthesize existing evidence on alterations in cognitive function among women with PMOS, compared to findings with healthy controls, and to identify gaps for future research.

## 2 METHODS

The review protocol was registered with PROSPERO (International Prospective Register of Systematic Reviews) (Centre for Reviews and Dissemination (CRD): CRD42024550999). The protocol and findings adhere to the Preferred Reporting Items for Systematic Reviews and Meta‐Analyses (PRISMA) guidelines (Table S1).^14^, ^15^ The criteria for inclusion for this review are outlined below.

### 2.1 Participants

Studies chosen for inclusion in the review will include participants diagnosed with PMOS, either through self‐report or through physician diagnosis.

### 2.2 Outcomes

The primary outcome of interest was cognitive function, and categorization was considered including memory or executive function, attention, attention switching, mental rotation, word recognition, and processing speed.

### 2.3 Exposure

The exposure of interest is a diagnosis or self‐report of PMOS prior to cognitive function testing.

### 2.4 Search strategy

A systematic search was performed on ovid MEDLINE, Embase, CINAHL, and PubMed databases for studies published from earliest availability to June 23, 2025. Search terms included combinations of the following medical subject headings and keywords: cognitive function, polycystic ovarian syndrome (outlined in Table S2). The search was conducted when the syndrome was referred to as polycystic ovarian syndrome. References of retrieved articles were also manually searched. The search strategy was developed in consultation with a health sciences librarian at Queen's University.

### 2.5 Study selection and extraction

Observational studies whose participants are patients who were diagnosed with PMOS and had their cognitive function evaluated after diagnosis were included in this review. The date of publication was not restricted given the limited literature available to answer the research question and the biological nature of the relationship that is unlikely to have changed over time. The studies chosen were restricted to those published in English.

Screening, full‐text review, and data extraction were facilitated using Covidence software.^16^ All stages of screening and data extraction were completed by both reviewers (SR and BM). The reference lists of included studies were reviewed for any additional missed publications. Disagreements were resolved through discussion. Cohen's kappa was calculated to measure inter‐rater reliability for full‐text review. The final selected articles underwent data extraction through Covidence, with detailed relevant information systematically extracted and input into Microsoft Excel. Variables extracted for each study related to the study characteristics (author, setting, and study dates), population demographics, details on exposure, and measures of association for cognitive functioning. Exposure was measured typically through Rotterdam diagnosis or self‐report; studies with either method of exposure measurement were eligible for inclusion in this review.

### 2.6 Synthesis methods

All meta‐analyses were performed in R Studio. To enable quantitative synthesis across studies using heterogeneous outcome metrics, all effect estimates were converted to a correlation coefficient (*r*) and then Fisher's *z* values (*zr*) with their associated standard errors (SE). SEs of Fisher's *z* were derived as SE(*zr*) = 1/√(*N* – 3), or directly from reported sampling variances when available. Fisher's *z* values were pooled under a random‐effects model (restricted maximum likelihood [REML] estimator) to account for between‐study heterogeneity.^17^, ^18^

Sensitivity analyses were conducted to assess heterogeneity using the Cochran's Q statistic and Galbraith plots and quantified using the *I* ^2^ statistic,^19^, ^20^ and studies were removed sequentially in each analysis to examine whether they contributed to heterogeneity.

### 2.7 Risk of bias assessment

Publication bias was visually examined using funnel plots.^21^ Quality of the studies was assessed using the Newcastle‐Ottawa Scale for cohort studies and modified for cross‐sectional studies.^22^

Ethics board approval for this study was not required.

## 3 RESULTS

### 3.1 Study selection

Using our defined search strategy, 2887 published studies were identified. After removing duplicates, non‐peer‐reviewed articles, and studies that did not meet our inclusion criteria or outcomes, 22 studies were included in the systematic review and meta‐analysis (Cohen's kappa = 0.98). Two articles were excluded from the meta‐analysis because they did not include a comparator group; however, they were included in the discussion.

### 3.2 Study characteristics

Across the 22 studies, 136,008 women with PMOS and 333,895 without PMOS were included. These studies are summarized in Table 1. Study designs were primarily cross‐sectional,^23^, ^24^, ^25^, ^26^, ^27^, ^28^, ^29^, ^30^, ^31^, ^32^ but also included case–control studies,^12^, ^13^, ^33^, ^34^, ^35^, ^36^, ^37^, ^38^ and cohort studies.^9^, ^11^, ^39^, ^40^, ^41^ Most studies assessed cognitive domains such as executive functioning, working memory, attention, and processing speed. A smaller number also investigated auditory and visual cognitive functions.

**TABLE 1:** Characteristics of included studies.

First author, year	Study design	Number of women in study	Age (mean;standard deviation (SD))	Location	Outcomes assessed	Main findings
Badariya, 2024	Cross‐sectional	60 (30 PMOS, 30 control)	Control: 22.3 years (1.57) PMOS: 22.0 years (1.56)	India	Working memory involved digit span and digit sequencing tasks tested working memory.	PMOS had lower mean scores overall with backward digit span, ascending sequence, descending sequence, visual digit span and sequencing tests. PMOS had overall lower scores and statistically significant different for the ascending sequence span tests.
Barnard 2007	Cross‐sectional	457,652 (135,215 PMOS, control 332,437)	PMOS without AA (anti‐androgen) medication: 30 (6.45) PMOS with AA: 29 (5.66) Control without AA medication: 31 (7.61) Control with AA medication: 26 (4.32)	United Kingdom	Mental rotation was the primary outcome of interest.	Performance did not differ according to diagnosis on mental rotation and spatial location tasks.
Barry, 2013	Cross‐sectional	110 (69 with PMOS, 41 controls)	PMOS mean age: 29 (18–38) Control mean age: 35 (24–43)	United Kingdom	Three‐dimensional mental rotation task.	PMOS scored significantly higher than control on 3D (three‐dimensional) mental rotation task.
Boivin, 2020	Case–control	120 (10 PMOS, 74 control, 25 screen positive)	PMOS: 29.09 (6.68) Control: 18.55 (0.89)	United States	Using Bilateral Field Advantage test, this study assessed response times.	Controls had faster response times and more correct responses for dots and letter presented bilaterally compared to women with PMOS, although these differences were not statistically significant.
Castellano, 2015	Case–control	18 (7 PMOS, 11 controls)	PMOS: 24.6 (5.9) Control: 24.0 (3.3)	Canada	Evaluation of working memory and attention was based on performance on the Paced Auditory Serial Addition Test (PASAT) and the Verbal Digit Span. The Trail Making and Stroop color word the Digit Symbol Substitution tests provided information on executive functions and processing speed.	The only assessed PMOS cognitive function scores to the normal range rather than the control group in the study, and thus could not be included in the meta‐analysis.
Ghazeeri, 2013	Case–control	37 (20 PMOS, 17 controls)	PMOS: 16.7 (1.1) Controls: 16.4 (1.3)	Lebanon	Inattention/hyperactivity was assessed as an outcome of interest.	*T*‐tests revealed significant differences between PMOS and control adolescent girls on hyperactivity scores (*p* = .025).
Herguner, 2015	Case–control	80 (40 PMOS, 40 controls)	PMOS: 22.28 (3.68) Control: 22.33 (3.66)	Turkey	Inattention/hyperactivity was assessed as an outcome of interest.	There was no significant difference between the PMOS group and the control. (*p* = .245).
Herguner, 2012	Case–control	80 (40 PMOS, 40 controls)	PMOS: 22.28 (3.68) Control: 22.58 (3.76)	Turkey	Outcomes of interest included attention.	There was no significant difference between the PMOS group and the control. (*p* = .200).
Huddleston, 2024	Prospective cohort	932 (66 PMOS, 866 controls)	PMOS: 54.7 (3.6) Controls: 55.3 (3.6)	United States	Montreal Cognitive Assessment (MoCA) was used attention, executive function, memory, language, visuospatial skills, calculations, and orientation. Processing speed was assessed with the Digit Symbol Substitution Test (DSST). Executive function was evaluated with the Stroop test.	At year 30, participants with PMOS performed lower (mean z score; 95% CI) on Stroop (−0.323 (−0.69 to −7.37); *p* = .008), RAVLT (−0.254 (−0.473 to −0.034); *p* = .002), and category fluency (−0.267 (−0.480 to −0.040); *p* = .02) tests.
Jarrett, 2019	Cross‐sectional	80 (40 PMOS, 40 controls)	PMOS: 27 (5.0) Control: 27 (4.0)	North America (United States and Canada)	Cognitive tests included working memory, including manual dexterity (Purdue pegboard task), perceptual speed (identical pictures task), and visuospatial ability (mental rotation task).	PMOS was independently associated with lower mental rotation task scores after adjustment for BMI (Body Mass Index). No evidence of an independent effect of PMOS on manual dexterity or perceptual speed was found.
Li, 2020	Retrospective cohort	82 (41 PMOS, 41 healthy controls)	PMOS: 25.29 (3.15), Controls: 26.22 (2.59)	China	The Two‐Back task was used to test working memory. The Stroop color word test was used to assess executive function.	Those with PMOS had impaired memory and executive functioning compared to those without PMOS; testing using the Two‐Back test was statistically significant whereas testing via the Stroop test was not significant.
Mehrabadi, 2020	Cross‐sectional	103 (53 PMOS, 50 Controls)	PMOS: 28.47 (6.27) Control: 29.94 (6.24)	Iran	The MoCA assesses six cognitive domains including executive functions; visuospatial abilities; short term memory; language; attention, concentration, and working memory; and temporal and spatial orientation.	Montreal Cognitive Assessments showed that some mean values were significantly lower in the women with PMOS compared to those without. Specifically, there were statistically significant differences in visual‐spatial ability, executive function, and attention scores.
Preeti, 2024	Cross‐sectional	50 (25 PMOS, 25 control)	PMOS: 20 (3.0) Controls: 18 (4.0)	India	Auditory reaction time and visual reaction time were assessed.	There were statically significant differences in auditory reaction time and visual reaction time between those with PMOS compared to without.
Redkar, 2024	Cross‐sectional	173 (101 PMOS, 72 controls)	PMOS: 25.75 (4.31) Control: 25.44 (3.79)	India	Focused attention (flanker task), divided attention (Posner Cueing task).	Reaction time for focused attention tasks found PMOS group took longer while control group responded faster, which was also observed for a divided attention task. Those with PMOS also made more errors in focused attention tasks while control group had higher accuracy and same trend with the divided attention task.
Rees, 2016	Cross‐sectional	36 (18 PMOS, 18 controls)	PMOS: 31 (6.0) Controls: 31 (7.0)	United Kingdom	Outcomes of interest included executive functioning tested by the Stroop color word test; and working memory tested via Digit Span. Letter and category fluency was also used to test executive function.	Those with PMOS had significantly lower scores on the Digit span forward test compared to controls (*p* = .02); all other results for outcomes of interest were not statistically significant.
Schattmann, 2007	Cross‐sectional	48 (28 PMOS, 20 controls)	PMOS: 27.7 (5.5) Control: 26.0 (6.0)	Canada	Digit Span tests was used to working memory; COWAT was used to test verbal fluency and mental rotation tests were also used.	Those with PMOS performed significantly worse than controls on tests of immediate memory, but were higher than controls on tests of working memory. On COWAT, those with PMOS group generated significantly fewer words then controls. Those with PMOS performed poorer on category fluency test, generating significantly fewer words then controls. PMOS performed worse than control on logical memory tests. Tests conducted using the Perdue pegboard found no differences in effect.
Showkath, 2022	Case–control	67 (37 PMOS, 30 controls)	PMOS: 25.07 (3.0) Controls: 24.38 (2.8)	India	The MoCA assesses six cognitive domains including executive functions; visuospatial abilities; short term memory; language; attention, concentration, and working memory; and temporal and spatial orientation.	The PMOS group had a significantly reduced total score of MoCA as well as all its components such as execution, attention, language, abstraction, memory, and orientation to time and place except naming, as compared to control subjects.
Soleman, 2016	Prospective cohort	34 (14 PMOS, 20 controls)	PMOS: 29.3 (5.6) Controls: 25.6 (6.2)	Netherlands/Belgium	Work memory was the outcome of interest, assessed via the Two‐Back test.	We found that women with PMOS differed in brain activation in the parietal lobe (superior and inferior) and in the temporal lobe (superior) than control women, but not in performance (number of errors and reaction time) during a working memory task.
Sukhapure, 2022a	Cross‐sectional	81 (40 PMOS, 41 controls)	All participants: 28.72	New Zealand	Digit Span tests was used to working memory.	Those with PMOS did not significantly different on the Digit Span test compared to those without PMOS (*p* = .89). There was no significant difference on COWAT scores between those with PMOS compared to without.
Sukhapure, 2022b	Prospective cohort	81 (40 PMOS, 41 controls)	PMOS: 29.24 (6.81) Controls: 28.20 (7.87)	New Zealand	Verbal learning memory, visuospatial learning and memory, psychomotor speed, executive function, verbal working memory and attention.	This study assessed correlation between testosterone levels and study outcomes of interest rather than control and PMOS groups, thus could not be included in the meta‐analysis.
Huddleston, 2022	Cross‐sectional	48 (all PMOS, 31 androgenic, 17 nonandrogenic phenotype)	PMOS with hyperandrogenism: 30.3 (5.9) PMOS without hyperandrogenism: 32.3 (4.3)	United States	Montreal Cognitive Assessment was used attention, executive function, memory, language, visuospatial skills, calculations, and orientation. Processing speed was assessed with the Digit Symbol Substitution Test (DSST). Executive function was evaluated with the Stroop test. Verbal memory was assessed with the Rey Auditory Verbal Learning Test (RAVLT)‐long delay. Verbal fluency was measured with the category and letter fluency tests.	The study only compared PMOS with hyperandrogenism to PMOS without hyperandrogenism and thus was not included in the meta‐analysis.
Franik, 2019	Case–control	55 with PMOS	All participants: 24.35 (4.16)	Poland	The Trail Making Test and Stroop test were used to assess attention, and the verbal fluency tests were used to assess executive functions.	Correlations were drawn between hormone levels and cognitive function, rather than comparing control and PMOS groups, thus, could not be included in the meta‐analysis.

Abbreviations: CI, confidence interval; COWAT, Controlled Oral Word Association Test; PMOS, polyendocrine metabolic ovarian syndrome.

### 3.3 Synthesis of results

The 22 studies that were included in the meta‐analysis are described in Table 1. The affected cognitive domains included attention, working memory, processing speed and accuracy, category fluency, and executive function.

### 3.4 Executive function

**FIGURE 1:**
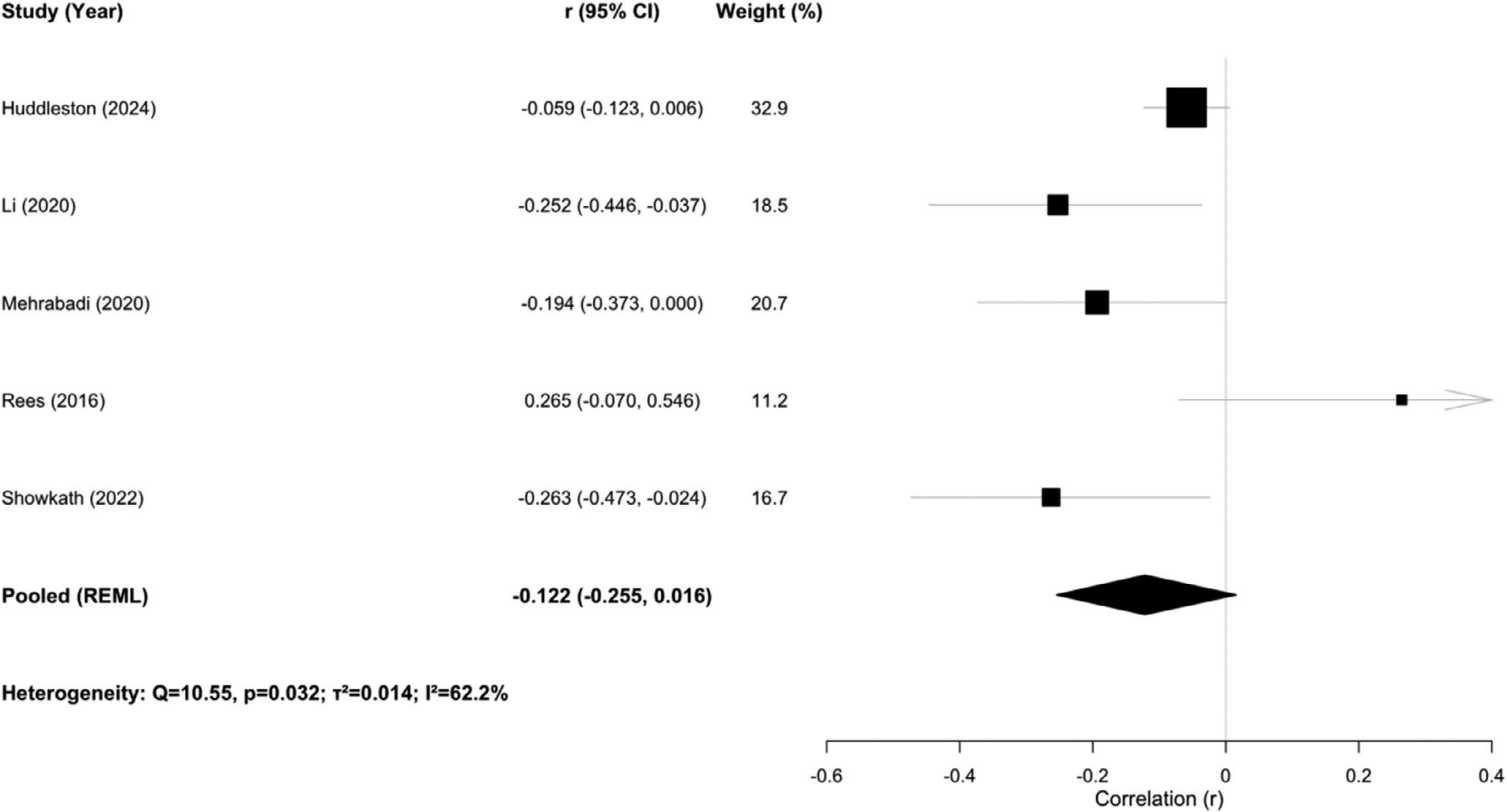
Meta‐analysis of differences in executive functioning in patients with polyendocrine metabolic ovarian syndrome (PMOS) compared to those without PMOS. CI, confidence interval; REML, restricted maximum likelihood.

Executive function was a commonly tested cognitive domain. The Trail Making Test and Stroop color word interference test, which measure psychomotor speed and cognitive flexibility,^42^ were used by five studies.^11^, ^27^, ^30^, ^37^, ^39^ The pooled correlation was −0.122 (95% confidence interval [CI]: −0.255, 0.016), which is a small, non‐significant negative association between PMOS and executive function performance (Figure 1). Heterogeneity was high (*I* ^2^ = 62.2%). Sensitivity analyses demonstrated that exclusion of Rees et al., reduced heterogeneity to 51.1%; however, removing other studies sequentially did not improve heterogeneity.

### 3.5 Attention

**FIGURE 2:**
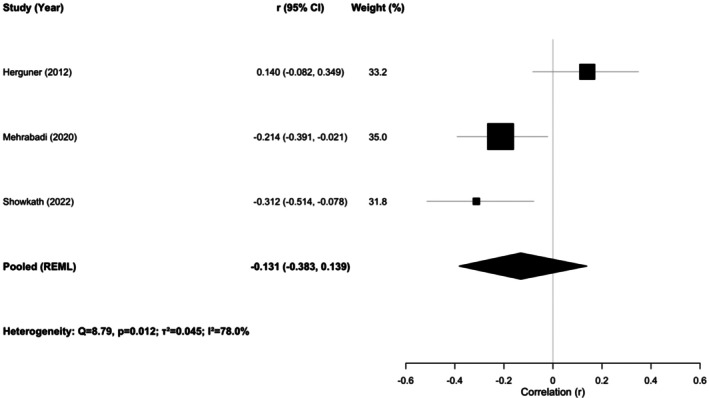
Meta‐analysis of attention in patients with polyendocrine metabolic ovarian syndrome (PMOS) compared to those without PMOS. CI, confidence interval; REML, restricted maximum likelihood.

Three studies assessed attention in individuals with PMOS compared to controls.^12^, ^27^, ^37^ The pooled correlation was −0.13 (95% CI: −0.38, 0.14), suggesting no significant association between PMOS and attention outcomes (Figure 2). However, there was evidence of substantial heterogeneity across studies (*I* ^2^ = 78.3%). Sensitivity analysis determined that heterogeneity was primarily driven by Herguner et al.^13^; exclusion of this study reduced heterogeneity to 0%, indicating that between‐study variability was explained by this single outlying result.

### 3.6 Working memory

**FIGURE 3:**
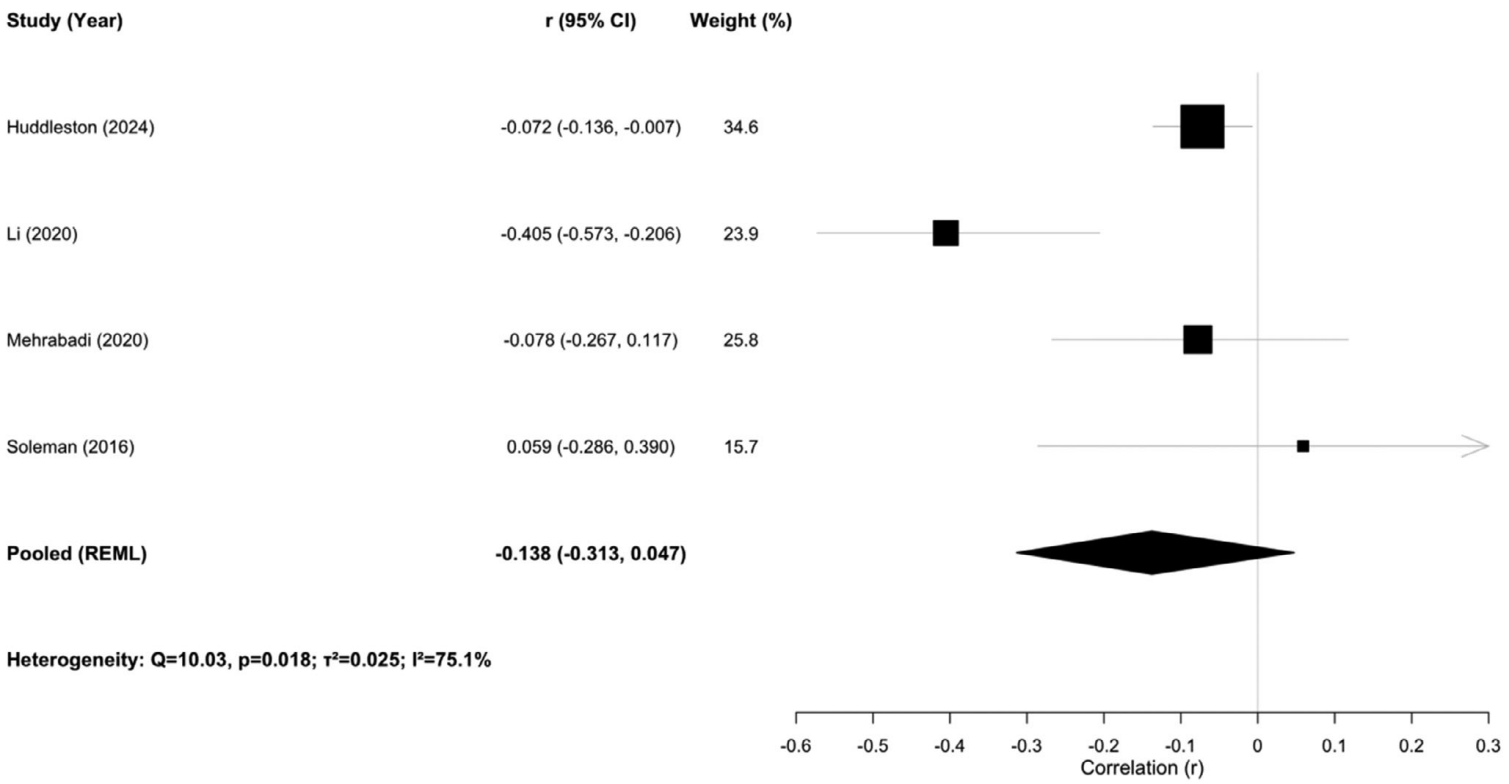
Meta‐analysis of working memory assessed via the Two‐Back Test in patients with polyendocrine metabolic ovarian syndrome compared to without. CI, confidence interval; REML, restricted maximum likelihood.

Four studies assessed working memory performance using the Two‐Back Test.^9^, ^11^, ^27^, ^39^ The pooled correlation was −0.138 (95% CI: −0.313, 0.047) under a random‐effects model, suggesting no significant association between PMOS and working memory (Figure 3). Substantial heterogeneity was observed (*I* ^2^ = 75.1%) and sensitivity analysis indicated that exclusion of Li et al. reduced heterogeneity to 0%, identifying this study as the main contributor to variability across results. Redkar and Khan reported similar findings using the Flanker and Posner Cueing tasks, with PMOS participants showing longer reaction times and reduced accuracy.^29^ This study was not included in the meta‐analysis as a different test was used and did not want to introduce additional heterogeneity.

Four studies evaluated visual backward digit span performance to measure working memory.^23^, ^30^, ^31^, ^32^ The pooled correlation was −0.066 (95% CI: −0.196, 0.067), indicating no significant association between PMOS and backward span outcomes (Figure S1). Heterogeneity was nonexistent (*I* ^2^ = 0.0%). These same four studies assessed visual forward digit span performance and found similar non‐significant results (−0.127; 95% CI: −0.348, 0.100)^23^, ^30^, ^31^, ^32^; however, there was considerable heterogeneity (*I* ^2^ = 64.2%) that was reduced to 0.0% when Schattmann was removed from the meta‐analysis (Figure S2).

### 3.7 Processing speed and reaction time

Three studies examined processing speed and reaction times; although they used different tests and thus were not combined in a meta‐analysis. Huddleston et al. used the Digit Symbol Substitution test (DSST) to assess processing speed and found no significant difference, but women with PMOS had a lower overall performance on a composite score.^11^ Preeti et al. found that women with PMOS demonstrated statistically significant longer auditory and visual reaction times compared to controls.^28^ Barnard et al. reported women with PMOS had impaired performance in accuracy and speed and overall slower reaction times on arrow tasks compared to the controls.^24^ Using Bilateral Field Advantage test, Boivin et al. reported that controls had faster response times and more correct responses for dots and letters presented bilaterally, compared to women with PMOS, although these differences were not statistically significant.^33^ However, this was the only study that conducted this test; thus, a meta‐analysis could not be performed.

### 3.8 Mental rotation

**FIGURE 4:**
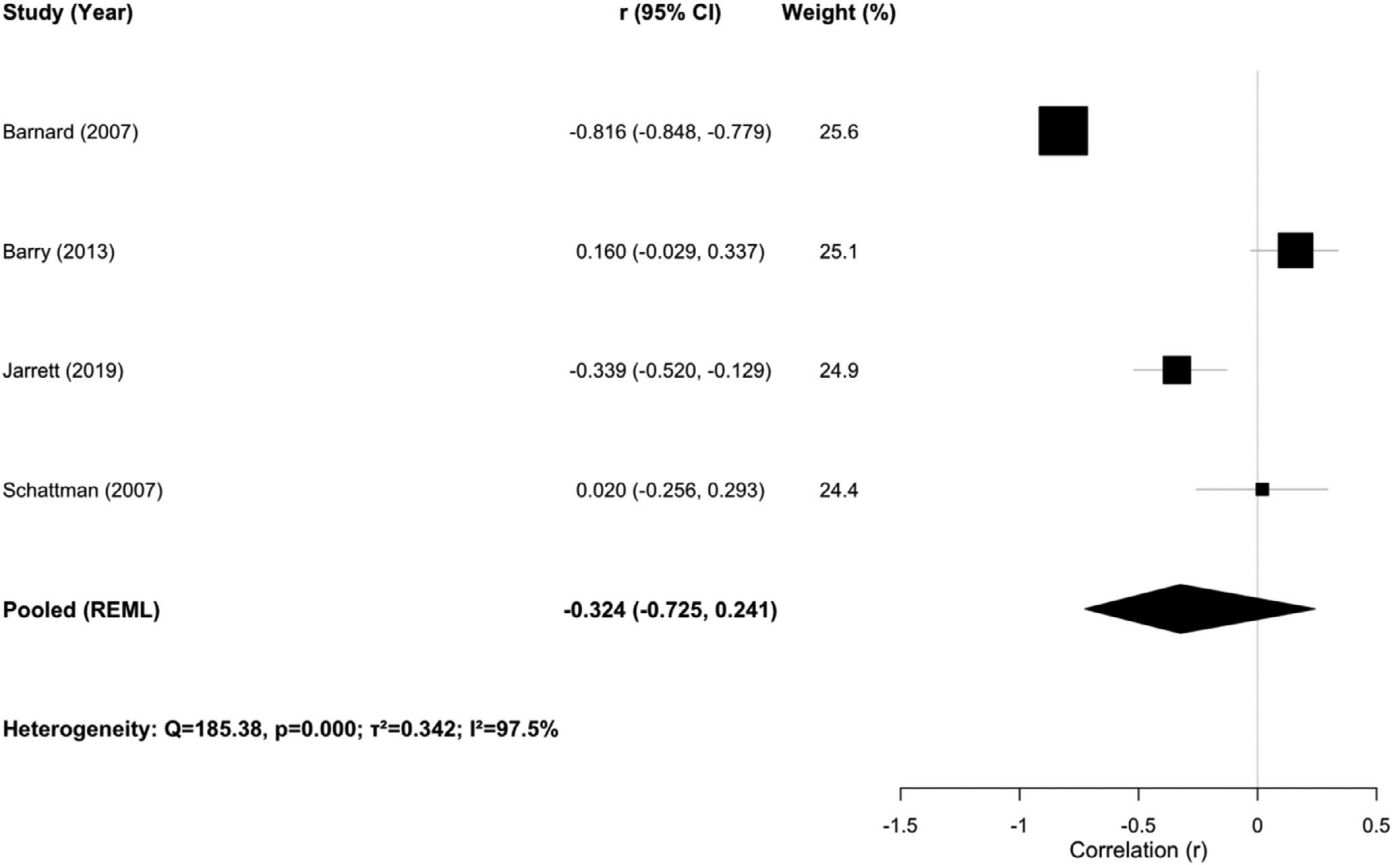
Meta‐analysis of differences in mental rotation in patients with polyendocrine metabolic ovarian syndrome compared to those without. CI, confidence interval; REML, restricted maximum likelihood.

Four studies examined mental rotation performance in individuals with PMOS compared to controls.^24^, ^25^, ^26^, ^31^ The pooled correlation was −0.324 (95% CI: −0.725, 0.241) (Figure 4), suggesting no significant association between PMOS and mental rotation ability. However, heterogeneity was very high (*I* ^2^ = 87.5%). Sensitivity analyses showed that sequential removal of individual studies did not reduce heterogeneity.

### 3.9 Auditory and sensory processing

Badariya and Surendran focused exclusively on the auditory and visual aspects of cognitive function and found that women with PMOS had significantly poorer mean scores in both ears than controls.^23^ On the Speech Perception In Noise (SPIN) test, the PMOS group exhibited lower mean scores, with statistically significant differences observed at 0 and −5 dB MCR (message to competition ratios), but not at +5 dB.

### 3.10 Verbal fluency

**FIGURE 5:**
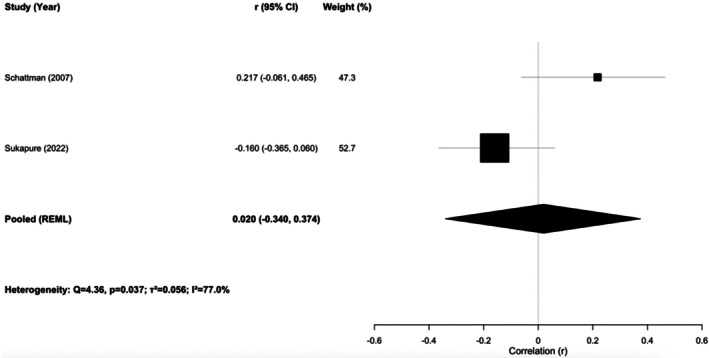
Meta‐analysis of differences in verbal fluency assessed via the Controlled Oral Word Association Test in patients with polyendocrine metabolic ovarian syndrome compared to those without. CI, confidence interval; REML, restricted maximum likelihood.

Two studies examined verbal fluency using the Controlled Oral Word Association Test (COWAT).^31^, ^32^ The pooled correlation was 0.020 (95% CI: −0.340, 0.374), indicating no significant association between PMOS and COWAT performance (Figure 5). Heterogeneity was substantial (*I* ^2^ = 77.0%). Sensitivity analyses suggested that sequential removal of individual studies did not resolve heterogeneity.

Verbal fluency was also assessed via category generation tasks.^11^, ^30^ The pooled correlation was not significant for this relationship (−0.001; 95% CI −0.064, 0.063) (Figure S3). There was no heterogeneity between these two studies (*I* ^2^ = 0.0%). Lastly, verbal fluency was assessed via letter generation tasks, and found similar non‐significant associations;^11^, ^30^ The pooled correlation was −0.129 (95% CI: −0.291, 0.048) and moderate heterogeneity was observed (*I* ^2^ = 34.6%), although no individual study was identified as a major driver of variability (Figure S4).

### 3.11 Risk of bias

There were no studies excluded due to risk of bias as outlined in Table S3. The only studies that were not included in the meta‐analysis were due to a lack of comparator, rather than bias. Generally, the studies were at low risk of bias, with only one study that had a high risk due to a lack of description of controls and their selection; however, this study was not included in the meta‐analysis.^28^

### 3.12 Publication bias

Funnel plots did not indicate any risk of potential publication bias for any outcomes. For example, the funnel plot for Executive Functioning demonstrates no risk of bias (Figure S5) (*b* = −0.1547 [CI: −0.7631, 0.4537]). Outcomes with only two included studies could not be assessed for publication bias.

## 4 DISCUSSION

This meta‐analysis is the first to focus on cognitive function in women with PMOS. While there were many studies that assessed various aspects of cognitive function, there were few that measured the same outcome using similar tests. Overall, our summary effects suggested no significant associations between a diagnosis of PMOS and cognitive functioning. A previous review of the literature described a consistent pattern of worse cognitive performance among those with PMOS compared to controls, specifically working memory, verbal fluency, and executive function; however, this study did not conduct a meta‐analysis on the findings.

There are four different phenotypes of PMOS and thus, there could be varying impacts on cognitive function due to different aetiologies of the condition. For example, Type D lacks a hyperandrogenic component.^43^ This is particularly important to consider as a possible reason for the discrepancies in outcomes, as it is the elevated androgens that are hypothesized to negatively impact cognition.^44^ Hyperandrogenism, insulin resistance, and chronic inflammation have been linked to cognitive aging and neurodegenerative risk in other populations.^45^ Yet, the young age of most participants in included studies, coupled with short follow‐up, may have limited the ability to detect these changes. It remains possible that cognitive effects of PMOS manifest later in life or only in subgroups characterized by more severe metabolic or hormonal disturbances. For example, hyperandrogenism has been proposed as one potential mechanism on the association between cognitive function and PMOS, although it remains unclear whether androgen excess directly affects cognition or is mediated through other pathways.^11^ Insulin resistance may also play an important role. Emerging evidence suggests that metabolic dysfunction may contribute more strongly to cognitive outcomes than androgen levels alone, with some studies reporting associations between markers of impaired glucose metabolism and poorer executive function, attention, and visuospatial abilities.^46^ Additionally, there is an increased risk of anxiety and depression for patients diagnosed with PMOS, which could potentially be a driver of impaired cognitive function.^44^, ^47^ Clinically, these results may help to reassure patients concerned about cognitive difficulties, while underscoring the importance of addressing comorbidities such as depression and anxiety.

### 4.1 Limitations

Heterogeneity in cognitive testing was substantial. Different instruments measure overlapping but not identical constructs, complicating pooled estimates. Tools used to measure similar outcomes also varied, which likely contributed to the high *I* ^2^ values observed in several analyses. Second, small sample sizes were common, with many studies likely underpowered to detect modest effects, increasing the risk of both false negatives and large CIs. Sensitivity analyses frequently identified single studies that disproportionately influenced pooled heterogeneity.

### 4.2 Conclusion

Although the number of studies assessing each outcome was relatively small, our findings suggest no association between a diagnosis of PMOS and impaired cognitive function for women in the age ranges assessed in the included studies. However, as there is an increased risk of anxiety and depression among these patients, there is still a need for increased monitoring on mental health and cognitive functioning.

## AUTHOR CONTRIBUTIONS

**Samantha Rogers:** Conceptualization; investigation; writing—original draft; writing—review and editing; formal analysis; data curation. **Jennifer Carpenter:** Supervision; project administration; writing—review and editing; resources; conceptualization; methodology; validation; data curation. **Bailey Milne:** Supervision; methodology; validation; writing—review and editing; investigation; conceptualization; software; formal analysis; visualization.

## CONFLICT OF INTEREST STATEMENT

The authors have nothing to disclose.

## ETHICS STATEMENT

Ethical approval was not required for this study.

## Supporting information


**Data S1.** Supporting Information.

## Data Availability

All data was taken from published literature.
