# Supplementary material for: Alterations in cognitive function among reproductive‐age women with polyendocrine metabolic ovarian syndrome: A systematic review and meta‐analysis
Source: J Neuroendocrinol. 2026 Jul 3;38(7):e70225. doi: 10.1111/jne.70225 (PMC13329973; doi:10.1111/jne.70225)

Supplemental Table 1. PRISMA Flowchart


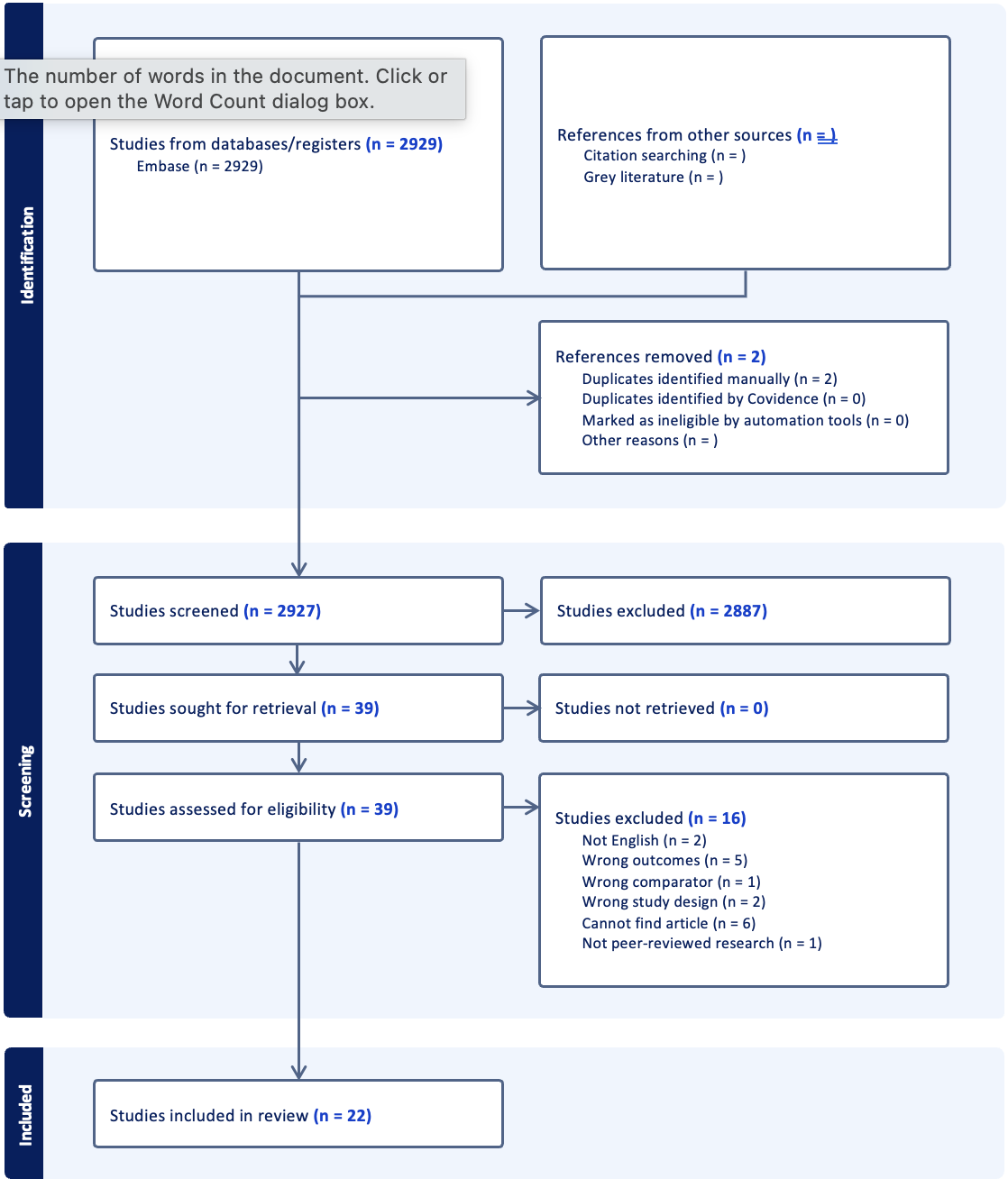


Supplemental Table 2. Search Strategy

| Search Number | Search Term |
| --- | --- |
| 1 | cognition/ or comprehension/ or processing speed/ or executive function/ or exp learning/ |
| 2 | exp Intelligence Tests/ |
| 3 | exp Neuropsychological Tests/ |
| 4 | Cognitive Dysfunction/ |
| 5 | Cognitive function.mp. |
| 6 | brain structure and function.mp. |
| 7 | IQ.mp. |
| 8 | Polycystic Ovary Syndrome/ |
| 9 | Polycystic ovary syndrome.mp. |
| 10 | pcos.mp. |
| 11 | cognitive assessment.mp. |
| 12 | Cognitive scores.mp. |
| 13 | Cognitive performance.mp. |
| 14 | Cerebral Activity.mp. |
| 15 | brain function change.mp. |
| 16 | (cognitive function and emotions).mp. [mp=title, book title, abstract, original title, name of substance word, subject heading word, floating sub-heading word, keyword heading word, organism supplementary concept word, protocol supplementary concept word, rare disease supplementary concept word, unique identifier, synonyms, population supplementary concept word, anatomy supplementary concept word] |
| 17 | executive functioning.mp. |
| 18 | memory.mp. or exp Memory/ |
| 19 | neuropsychological evaluation scores.mp. |
| 20 | executive function impairments.mp. |
| 21 | brain functional abnormalities.mp. |
| 22 | working memory.mp. |
| 23 | Task performance.mp. |
| 24 | (speed and accuracy).mp. [mp=title, book title, abstract, original title, name of substance word, subject heading word, floating sub-heading word, keyword heading word, organism supplementary concept word, protocol supplementary concept word, rare disease supplementary concept word, unique identifier, synonyms, population supplementary concept word, anatomy supplementary concept word] |
| 25 | overall brain activity.mp. |
| 26 | memory load condition.mp. |
| 27 | executive functioning.mp. |
| 28 | 1 or 2 or 3 or 4 or 5 or 6 or 7 or 11 or 12 or 13 or 14 or 15 or 16 or 17 or 18 or 19 or 20 or 21 or 22 or 23 or 24 or 25 or 26 or 27 |
| 29 | Endocrine disorder.mp. |
| 30 | 8 or 9 or 10 or 29 |
| 31 | 28 and 30 |

Supplemental Table 3. Risk of Bias Analysis

| **Study** | **Representativeness of the exposed cohort** | **Selection of the non exposed cohort** | **Ascertainment of exposure** | **Demonstration that outcome of interest was not present at the start of the study** | **Comparability of cohorts on the basis of the disease or analysis** | **Assessment of outcome** | **Was follow-up long enough for outcomes to occur** | **Adequacy of follow up of cohorts** | **Score** |
| --- | --- | --- | --- | --- | --- | --- | --- | --- | --- |
| Huddleston 2024 | * | * | * | * | ** | * | * | * | 9 |
| **Study** | **Is the case definition adequate?** | **Representativeness of the cases** | **Selection of controls** | **Definition of controls** | **Comparability of cases and controls on the basis of the design or analysis** | **Ascertainment of exposure** | **Same method of ascertainment for cases and controls** | **Non-response rate** | **Score** |
| Badariya 2024 | * |  | * |  | * | * | * | * | 7 |
| Barnard 2007 | * | * | * |  | ** |  | * |  | 6 |
| Barry 2013 | * | * |  | * | * | * | * | * | 8 |
| Boivin 2020 | * |  | * | * | ** | * | * |  | 7 |
| Castellano 2015 | * |  |  | * |  | * | * |  | 4 |
| Ghazeeri 2013 | * |  |  | * | ** | * | * |  | 6 |
| Herguner 2015 | * | * |  | * | ** | * |  |  | 6 |
| Herguner 2012 | * | * | * | * | ** | * |  |  | 7 |
| Jarrett 2019 | * |  | * | * | * | * | * |  | 6 |
| Li 2020 | * |  | * | * | ** | * |  |  | 6 |
| Mehrabadi 2020 | * | * |  |  | ** | * |  |  | 5 |
| Preeti 2024 | * |  |  |  |  |  | * |  | 2 |
| Redkar 2024 | * | * |  | * | ** | * |  |  | 6 |
| Rees 2016 | * | * |  | * | ** | * | * |  | 7 |
| Schattman 2007 | * | * | * | * | ** | * | * | * | 9 |
| Showkath 2022 | * | * | * |  | ** | * |  |  | 6 |
| Soleman 2016 | * | * | * | * | ** | * | * |  | 8 |
| Sukhapure 2022a | * | * | * | * | ** | * | * |  | 8 |
| Sukhapure 2022b | * | * | * | * | ** | * | * |  | 8 |

Supplemental Figure 1. Meta analysis of working memory assessed via Visual Backward Digit Span for patients with PMOS compared to without


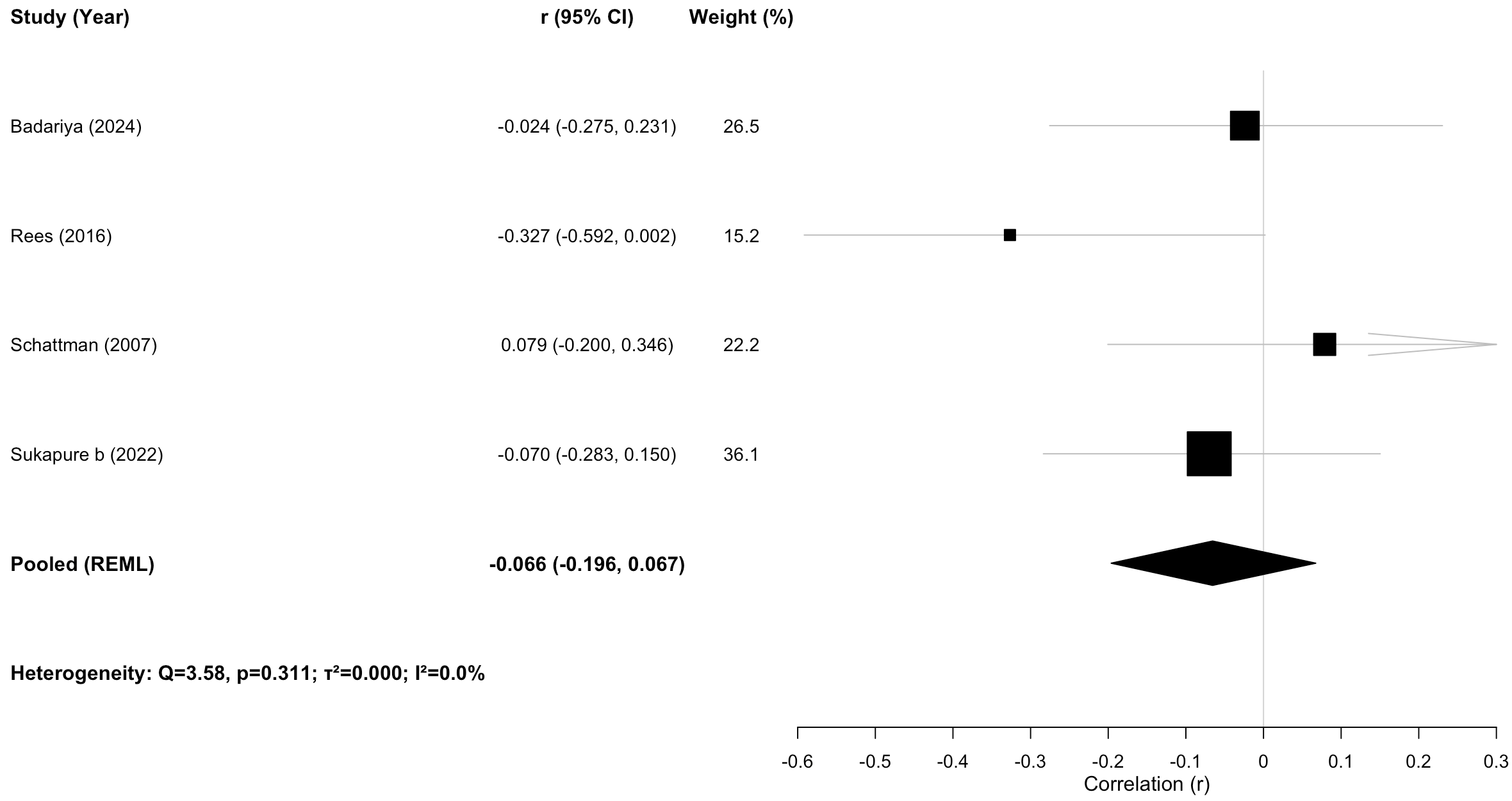


Supplemental Figure 2. Meta analysis of working memory assessed via Visual Forward Digit Span for patients with PMOS compared to without


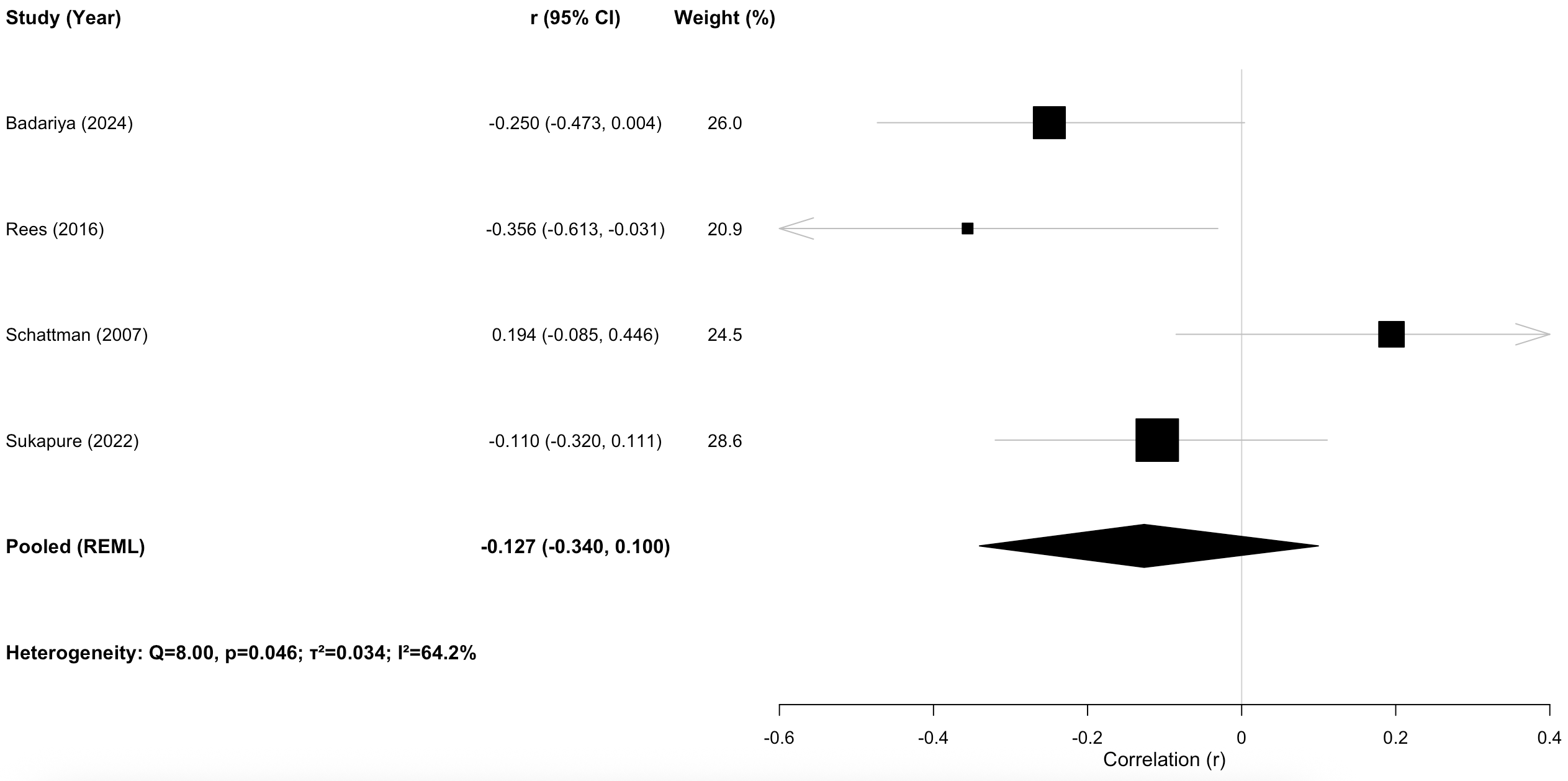


Supplemental Figure 3. Meta analysis of verbal fluency assessed via category generation tasks for patients with PMOS compared to without


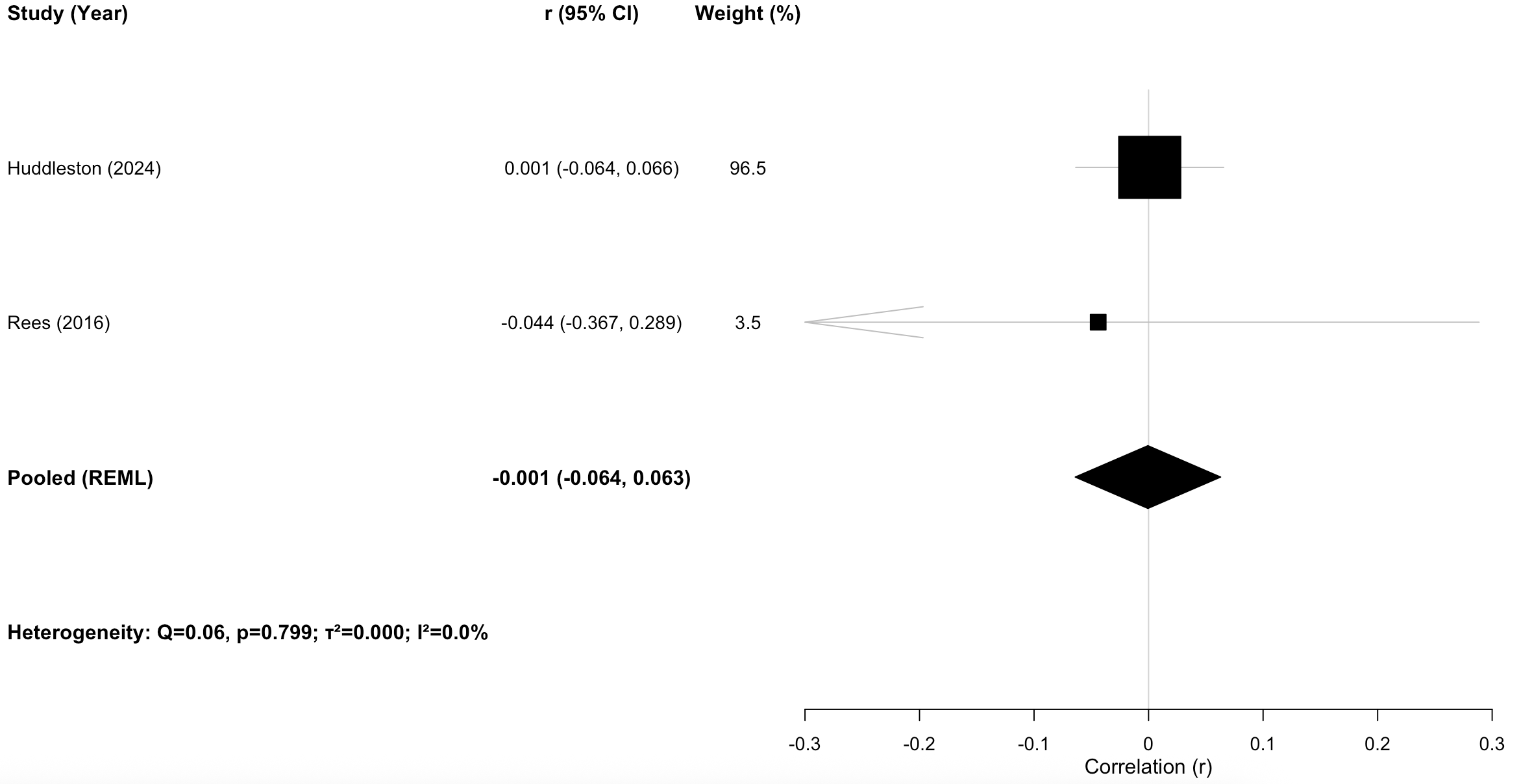


Supplemental Figure 4. Meta analysis of verbal fluency assessed via letter generation tasks for patients with PMOS compared to without


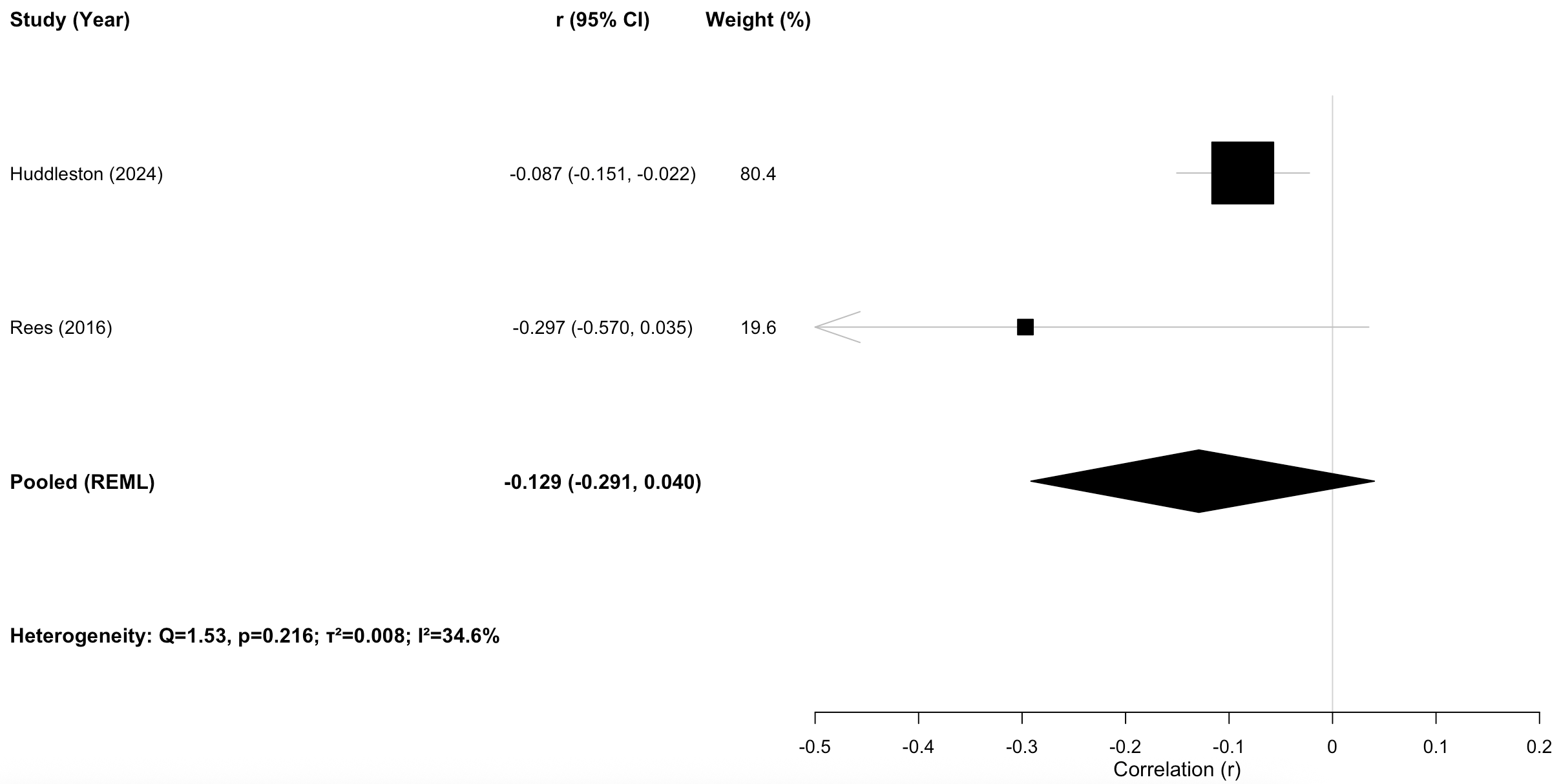


Supplemental Figure 5. Funnel plot for publication bias for studies that assessed executive function


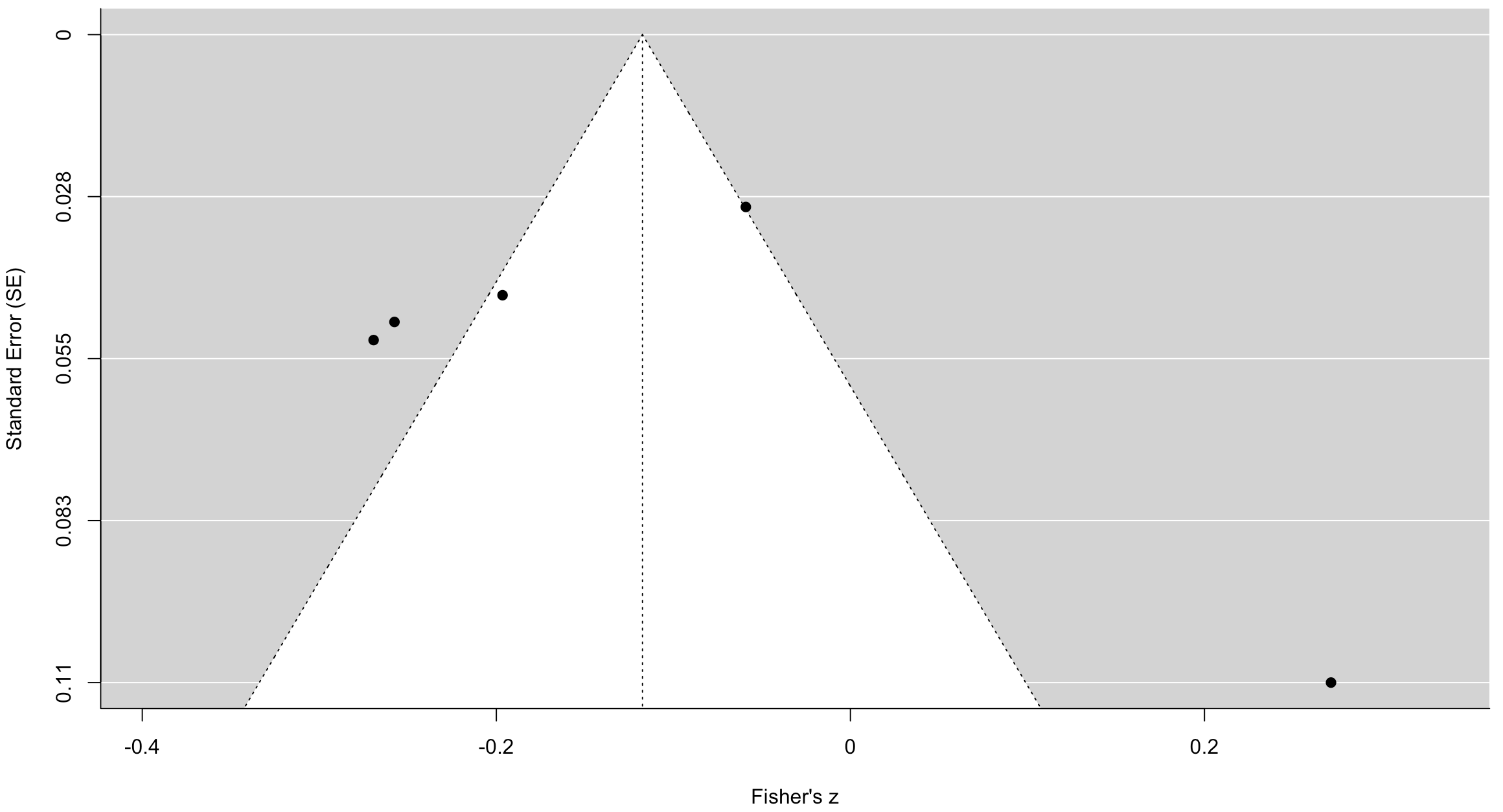

Supplement: Supplementary file 1 — Data S1. Supporting Information. [file JNE-38-e70225-s001.docx]
